# Vascular Endothelial Growth Factor Receptors in the Vascularization of Pancreatic Tumors: Implications for Prognosis and Therapy

**DOI:** 10.3390/cimb47030179

**Published:** 2025-03-10

**Authors:** Craig Grobbelaar, Vanessa Steenkamp, Peace Mabeta

**Affiliations:** 1 Department of Physiology, School of Medicine, Faculty of Health Sciences, University of Pretoria, Pretoria 0002, South Africa; peace.mabeta@up.ac.za; 2 Department of Pharmacology, School of Medicine, Faculty of Health Sciences, University of Pretoria, Pretoria 0002, South Africa; vanessa.steenkamp@up.ac.za

**Keywords:** VEGFR-1, VEGFR-2, VEBFR-3, pancreatic cancer, angiogenesis, lymphangiogenesis, metastasis, hypoxia, tumor microenvironment, vascular homeostasis

## Abstract

In pancreatic cancer (PC), vascular endothelial growth factor (VEGF) and its primary receptor, vascular endothelial growth factor receptor (VEGFR)-2, are central drivers of angiogenesis and metastasis, with their overexpression strongly associated with poor prognosis. In some PC patients, VEGF levels correlate with disease stage, tumor burden, and survival outcomes. However, therapies targeting VEGF and VEGFR-2, including tyrosine kinase inhibitors (TKIs) and monoclonal antibodies, have demonstrated limited efficacy, partly due to the emergence of resistance mechanisms. Resistance appears to stem from the activation of alternative vascularization pathways. This review explores the multifaceted roles of VEGFRs in pancreatic cancer, including VEGFR-1 and VEGFR-3. Potential strategies to improve VEGFR-targeting therapies, such as combination treatments, the development of more selective inhibitors, and the use of biomarkers, are discussed as promising approaches to enhance treatment efficacy and outcomes.

## 1. Introduction

Pancreatic cancer (PC) is an aggressive malignancy with poor survival rates and limited treatment options [1]. This cancer has alarmingly high incidence and mortality rates, affecting both high-income and low-to-middle-income countries. According to GLOBOCAN 2022, PC is the 12th most commonly diagnosed cancer worldwide, with an estimated 510,992 new cases and an age-standardized incidence rate of 4.7 per 100,000 population. It is also the 6th leading cause of cancer-related mortality, accounting for 467,409 deaths globally [2]. Approximately 90% of PCs are pancreatic ductal adenocarcinomas (PDAC) [3]. Pancreatic neuroendocrine tumors (PNETs) are the second most common form of PC [3]. Angiogenesis-promoting molecules are overexpressed in both PDAC and PNET, contribute to low survival rates, and are associated with the development of resistance to chemotherapy and immunotherapy [3]. Furthermore, aggressive angiogenesis is associated with malignant PNET [4]. Angiogenesis, the formation of blood vessels from pre-existing microvessels, is necessary for the growth of various tumors, as well as their malignant transformation, and is regulated by pro- and anti-angiogenic molecules [4,5]. Of these regulators, vascular endothelial growth factor-A (referred to as VEGF) is the most studied.

VEGF is the first angiogenic factor that was isolated and sequenced; subsequently, several dimeric proteins structurally related to VEGF were identified. The dimeric molecules collectively constitute the VEGF family and exert their effects through binding to vascular endothelial growth factor receptors (VEGFRs) [5]. These receptor tyrosine kinases (RTKs) share structural similarities [4,5,6]. VEGFRs have three domains: an extracellular domain with seven immunoglobulin-like domains that bind to a ligand, a single transmembrane helix that anchors the protein, and a split intracellular domain that plays a catalytic role [7,8]. RTKs catalyze the transfer of a phosphoryl group from a nucleotide (commonly adenosine triphosphate-ATP) to the tyrosine residue of a substrate molecule, leading to the activation of downstream effectors [8]. The aberrant expression of VEGFRs is associated with several malignancies [6]. As a result, tyrosine kinase inhibitors (TKIs), which target mainly VEGFR-2, have been developed to curtail tumor angiogenesis [9]. The use of these inhibitors is associated with relapse partly due to the activation of alternative pathways involving other VEGF family ligands and receptors, and thus, an understanding of the molecular biology of VEGFRs, as well as their pathophysiological roles, is important to improve cancer therapy. Various VEGFR variants have been identified in recent years [6]. These variants have physiological roles distinct from their membrane-anchored counterparts [6]. In addition, some isoforms are associated with poor prognosis in several cancers [6]. Hence, the potential roles of VEGFR variants in PC therapy have relevance. This review provides a narrative synthesis of the current literature on VEGF/VEGFR in PC, focusing on recent findings and emerging therapeutic strategies. Referenced articles were selected based on their relevance to VEGFR signaling, PC pathophysiology, and therapeutic implications, with preference given to recent peer-reviewed studies, clinical trials, and high-impact reviews. While previous reviews have broadly examined VEGF/VEGFR signaling and therapeutic strategies in oncology [10,11,12,13], this review examines explicitly the distinct roles of VEGFR-1, -2, and -3 in PC, emphasizing their contributions to tumor progression, therapeutic resistance, and the limitations of VEGFR treatment in the most common forms of PC. Novel inhibitors, resistance mechanisms, and the exploitation of VEGFR variants for improved patient care, particularly in treatment and prognosis, are highlighted. Additionally, this review explores the role of predictive biomarkers and combination strategies that may enhance the efficacy of VEGFR-targeted therapies in PC.

## 2. VEGF Receptors and Their Ligands

Receptor tyrosine kinases are classified according to similarities in their sequences. VEGF receptors belong to the class V RTKs due to the conserved 16 cysteine residues in their extracellular domain [9,14]. This class of receptors mainly binds to the VEGF family of ligands, which constitutes (i) vascular endothelial growth factor receptor-1 (VEGFR-1) or Fms-like tyrosine kinase (Flt-1), (ii) vascular endothelial growth factor receptor-2 (VEGFR-2), also referred to as kinase insert domain-containing receptor (KDR), and (iii) vascular endothelial growth factor receptor-3 (VEGFR-3), also known as Fms-related receptor tyrosine kinase 4 (Flt-4) [4,8,14]. Advancements in protein detection methods have enhanced sensitivity, shedding light on the expression patterns of these receptors in various cancers, including PC.

### 2.1. VEGFR-1 Enables Pancreatic Cancer Vascularization

VEGFR-1 is mainly expressed in blood vascular endothelial cells (ECs), hematopoietic monocytes, and macrophages [14,15]. The receptor was initially identified in the lungs and later in other highly vascularized tissues; it has also been identified in various cancers [14]. VEGFR-1 was detected in several PC cell lines and localized on PC tissue. In PC cells, VEGFR-1 signaling promotes proliferation and migration [16]. In vivo, signaling through the receptor contributes to PC progression. VEGFR-1 binds VEGF with high affinity but has low kinase activity due to the absence of phosphorylation of the regulatory kinase residue [15,16]. The suppression of phosphatidylinositol-3 kinase (PI3k) activation by the transmembrane helix when the receptor binds to VEGF has also been observed [4,8,14]. These observations further support the assertion that VEGFR-1 has a considerably low mitogenic effect when transducing through VEGF.

**Figure 1:**
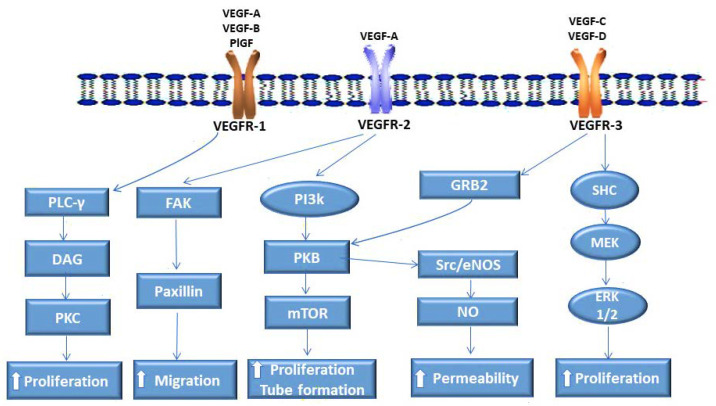
Diagram indicating vascular endothelial growth factor receptor (VEGFR) signaling in pancreatic cancer. VEGFR-1 binds VEGF-A, Placental Growth Factor (PlGF), and VEGF-B to regulate angiogenesis. VEGFR-2 binds VEGF-A to primarily drive angiogenesis. VEGFR-3 binds VEGF-C and VEGF-D to elicit lymphangiogenesis. Key signaling pathways involved include extracellular signal-regulated kinase (ERK), phosphoinositide 3-kinase (PI3K), and protein kinase C (PKC). The figure was constructed using drawing tools and Sketch.

Two ligands belonging to the VEGF superfamily, vascular endothelial growth factor-B (VEGF-B) and placental growth factor (PlGF), bind exclusively to VEGFR-1 (Figure 1) [8,15]. The deletion of the VEGFR-1 gene results in the mortality of mouse embryos [17]. This is attributable to the absence of the regulatory effect of this protein on VEGF/VEGFR-2 signaling. In mice that lack only the intracellular domain of VEGFR-1, the vasculature develops normally [17]. Noteworthy is that the exclusive ligands for VEGFR-1, PlGF, and VEGF-B are not essential for the development of the vasculature in the embryo [16,18,19,20]. The implication is that VEGFR-1’s importance in embryonic angiogenesis is largely due to its role as a negative regulator of VEGF/VEGFR-2 signaling, which is regulated by the intracellular domain of the receptor. Although in the normal physiological setting, VEGFR-1 binds to VEGF with an affinity that is approximately 10-fold higher than that of VEGFR-2, most of the ligand’s angiogenic activity is a result of its activation of VEGFR-2 [9,18,19]. Noteworthy is that the exclusive ligands for VEGFR-1, PlGF, and VEGF-B are not essential for the development of the vasculature in the embryo [16,18,19,20]. In contrast, PlGF is highly expressed in PC, and VEGFR-1/PlGF plays an important role in PC cell proliferation and migration [20]. In neoplastic disease, the binding of VEGFR-1 to PlGF results in a more pronounced mitogenic activity in ECs and further promotes cell migration and invasion [4,5,7,14]. PlGF promotes pathological angiogenesis in PC patients, and high levels of the ligand correlate with the disease stage in PNET [20]. Important to note is that studies have shown that VEGFR-1/PlGF is dispensable in adult angiogenesis [21]. From these observations, VEGFR-1/PlGF targeting could be of clinical benefit in neoplasms such as PC, which have shown a poor response to VEGFR-2 targeting approaches and are associated with aberrant VEGFR-1/PlGF signaling.

### 2.2. VEGFR-2 Plays a Pivotal Role in Pancreatic Cancer Angiogenesis

VEGFR-2, primarily activated by VEGF, is the principal mediator of angiogenesis and is widely distributed and expressed in all vessel-derived endothelial cells. This class III transmembrane protein kinase is aberrantly expressed in various tumors [22]. PNET, a hypervascularized form of PC, is characterized by high expression levels of VEGFR-2 [23]. The most common form of PC, PDAC, is not highly vascularized, but it exhibits enhanced VEGFR-2 expression. VEGF-A/VEGFR-2 signaling has been identified as a key pathway in the growth and metastasis of both forms of PC [24]. Additionally, over-expression of VEGF as well as VEGFR-2 is associated with poor prognosis as well as resistance to anti-angiogenic treatments in PDAC [25]. Notably, aberrant activation of VEGFR-2 by VEGF supports both paracrine angiogenic mechanisms and autocrine mitogenic pathways in PC cells. In the PC stroma, paracrine interaction of VEGFR-2/VEGF promotes endothelial cell proliferation, survival, and neovascularization via downstream signaling of mitogen-activated protein kinase (MAPK)/protein kinase C (PKC)/PI3K (Figure 1) [26]. VEGFR-2/VEGF also functions upstream of the DLL4/Notch pathway by regulating tip cell dynamics and coordinating angiogenic sprouting [10,27]. Furthermore, VEGFR-2 influences other aspects of tumor biology, including enhanced cancer cell migration, invasion, and resistance to apoptosis, thereby contributing to the aggressive nature of PC. VEGFR-2 positivity has been reported in 69% of PC cell lines, and in PC patients, it correlates with poor prognosis and serves as an independent predictive factor for aggressive disease [10]. VEGFR-2 promotes cancer stem cell proliferation and self-renewal through the VEGF/Neuropilin-1 and VEGFR-2/STAT3 pathways [28,29,30]. Neuropilin-1 and -2, which act as co-receptors for VEGF-A, form complexes with VEGFR-1 and -2, thereby promoting angiogenesis and aberrant signaling in PC [28,29,30]. Additionally, VEGF/VEGFR-2 suppresses T cell maturation, impairs the maturation of dendritic cells (DCs), and induces their differentiation into EC-like cells [31]. Thus, VEGFR-2 signaling modulates the pancreatic tumor microenvironment by creating an immunosuppressive milieu. VEGFR-2 can bind to VEGF-C/D, albeit with much less potency than VEGFR-3 [10,15]. Therefore, beyond angiogenesis, VEGFR-2 activity might extend to the promotion of vascularization through other pathways, highlighting its multifaceted role in PC progression and presenting opportunities for combination therapeutic intervention to curtail metastasis.

### 2.3. VEGFR-3 in the Lymphatic Dissemination of Pancreatic Cancer

VEGFR-3 mainly binds to VEGF-C and VEGF-D (Figure 1) [32,33]. The receptor is mostly expressed in lymphatic endothelial cells (LECs), macrophages, and monocytes, as well as fenestrated veins and capillaries of endocrine organs [34,35,36]. The ligand for VEGFR-3, VEGF-C, is overexpressed in human PC [37]. Interestingly, PC is one of the first human malignancies that demonstrated VEGF-C-associated lymphangiogenesis [38]. VEGF-C signaling through VEGFR-3 involves the activation of Cdc-related kinase (CRK) I/II and C-Jun N-terminal kinase (JNK) 1/2 [7,8,34]. The binding of VEGF-C and -D to VEGFR-3 can also activate PI3k and its downstream effector PKB (Figure 1) [7,35,39]. The activation of PI3k induces LEC proliferation [35,39]. Another pathway that can be activated following the binding of VEGFR-3 to its major ligands is growth factor receptor binding protein 2 (GRB2), ultimately leading to the activation of extra-cellular regulated kinase (ERK)1/2 [4,7,39]. Such activation of the ERK pathways promotes lymphatic vessel formation [34,35,36,39]. 

Postnatally, VEGFR-3 remains a major receptor in the regulation of lymphangiogenesis, which is the formation of lymphatic vessels from already existing lymphatics [38]. It is noteworthy that the receptor also modulates lymphangiogenesis in various pathologies, including PC [35,36,39,40,41,42]. In PC, VEGFR-3 is localized to both the neoplastic cells and the tumor endothelial cells (TECs) [36,39,40,41]. VEGFR-3 binds to another VEGF-family molecule, VEGF-D, which was found to be overexpressed in up to 36% of PCs [43,44,45]. Importantly, there is a significant correlation between VEGF-D/VEGFR-3 activation, lymphatic metastasis, and poor patient outcomes [46]. These observations highlight the importance of VEGFR-3 signaling in disease progression in PC, although studies exploring this relationship and its possible role in PC therapy remain sparse.

## 3. VEGF Receptor Targeting in Pancreatic Cancer

The abnormal expression of VEGF receptors has been observed in multiple neoplasms and, in some instances, correlates with resistance to cancer treatment [14,29,40,41,42]. While VEGFR-2 has been reported extensively, VEGFR-1 and -3 have received far less attention. The latter two receptors are thus covered more in-depth in this review. Moreover, the clinical development of anti-angiogenic receptor inhibitors has largely focused on VEGFR-2 targeting. Disappointingly, the outcome of VEGFR-2-directed monotherapy in cancer patients has been modest [5,9,22]. To improve the efficacy of tumor angiogenesis suppression, research has since focused on combination approaches that target other RTKs in addition to KDR, including VEGFR-1 and VEGFR-3.

### Recent Developments in VEGFR Targeting in Pancreatic Cancer

VEGFR-1 targeting has mainly employed kinase inhibitors that also target VEGFR-2 [47]. These angiogenesis inhibitors were developed to target the VEGF receptors in combination with other class V RTKs (Table 1). Recently developed drugs that target VEGFRs mainly belong to types I, II, or III kinase inhibitors based on their mode of action [48]. Type I inhibitors bind to the active ATP site, while type II inhibitors target the inactive conformation, thus conferring a greater degree of selectivity, whereas type III TKIs bind remotely from the ATP binding site [48]. These drugs have been employed to treat various forms of PC (Table 1).

Antiangiogenic drugs developed and approved by various regulatory bodies in the last decade include lenvatinib, a type II TKI approved for treating hepatocellular (HCC), renal, thyroid, and endometrial cancers (Table 1) [49]. Lenvatinib, which inhibits VEGFR-1, -2, and -3 signaling, has also been evaluated in phase II clinical trials for treating advanced PNET, highlighting its potential application in this challenging cancer type [49]. Concerning the drug’s effects and mechanism in experimental models, it inhibits angiogenesis and tumor cell growth in mouse xenografts of various cancers [58]. Lenvatinib also normalizes tumor blood vessels [58]. Its side-effect profile warrants the employment of delivery systems that will minimize off-target effects. Regorafenib is another type II TKI that targets all three VEGFRs, as well as KIT, and inhibits neovessel growth and tumor progression. It was approved in the same year as lenvatinib for treating gastrointestinal (GI), hepatocellular carcinoma, and colorectal cancer [59,60]. Regorafenib has shown promising results in several tumors and could be useful in combination approaches for treating tumors resistant to VEGF/VEGFR-2 inhibitors [60]. However, despite its efficacy in other cancers, a phase II trial of regorafenib in patients with PDAC failed to meet its primary endpoint with only a 25% progression-free survival (PFS) rate at 8 weeks and a median progression-free survival of 1.7 months. The investigators concluded that further evaluation of regorafenib monotherapy in PC is not warranted [50] (Table 1).

Cabozantinib, a multikinase inhibitor, targets VEGFR-1, -2-, and -3, MET, and AXL to inhibit angiogenesis, tumor growth, and metastasis. It has demonstrated efficacy in PNETs, significantly prolonging PFS in the phase III CABINET trial, and its use in PNETs is currently under the U.S. Food and Drug Administration (FDA) evaluation due to these promising results [51]. Cediranib, a small molecule inhibitor with antiangiogenic properties, was evaluated in combination with durvalumab (anti-PD-L1 immunotherapy) in patients with PDAC as part of the DAPPER study (phase II trial). The trial demonstrated limited antitumor activity, with only one patient achieving an unconfirmed partial response and 16 of 18 evaluable patients experiencing disease progression [52]. Chen et al. demonstrated that another anti-angiogenic drug, apatinib, which mainly targets VEGFR-2, did not achieve statistical significance across all endpoints when administered in combination with camrelizumab (an anti-PD-1 immune checkpoint inhibitor) [57]. However, there was improved survival and disease control, suggesting this combination may be a viable option for previously treated metastatic PDAC [53]. In a phase III study where the effect of axitinib (VEGFR-1, -2, and -3 inhibitor) plus gemcitabine was compared to the placebo plus gemcitabine in patients with advanced PDAC, no significant improvement in overall survival for the axitinib group compared to the placebo group was noted, which is indicative thereof that axitinib did not enhance the efficacy of gemcitabine in this cohort [56]. Other multi-targeting TKIs, including sunitinib and sorafenib, have been evaluated in patients with PC. Interestingly, sunitinib has been approved for use in locally advanced or metastatic PNETs since 2011. Despite promise in earlier studies, the addition of sorafenib did not provide a therapeutic benefit over gemcitabine alone in patients with advanced PC [55]. These multi-targeting TKIs are mainly designed to inhibit angiogenesis and normalize tumor blood vessels. However, given the limitations of these TKIs in PC, which have been highlighted in this review and in other studies, and the complex regulation of VEGFRs through alternative splicing, VEGFR splice variants warrant consideration in anti-angiogenic therapeutic strategies.

## 4. VEGFR-1, -2, and -3 Variants in Pancreatic Cancer Therapy

The modest success of VEGF-neutralizing approaches in cancer treatment led to the emergence of therapeutics targeting VEGFR-2, including TKIs [61,62]. These therapeutic molecules have shown limited clinical success [62,63,64]. In the last decade, drug development has been directed at refining existing VEGFR targeting approaches, including developing therapies that modulate splicing events or selectively inhibit specific VEGFR isoforms.

### 4.1. Regulation of VEGFRs Through Alternative Splicing

**Figure 2:**
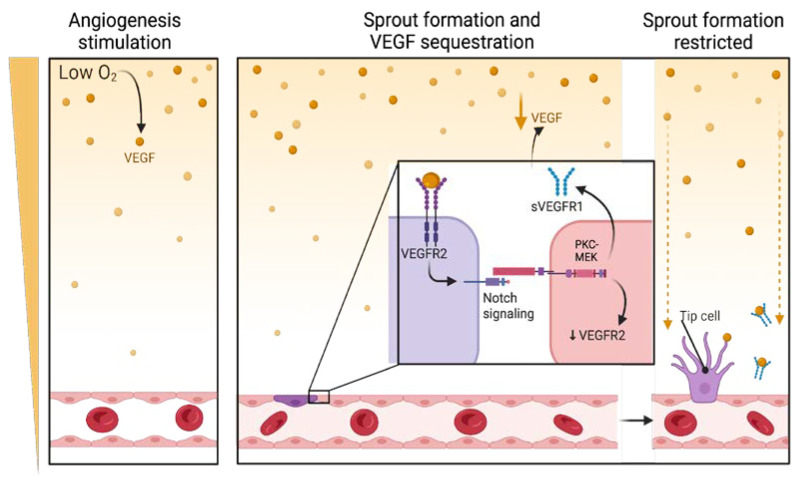
Diagram indicating how vascular endothelial growth factors receptor-1 and -2 regulate angiogenesis. VEGF-VEGFR-2 activates Notch signaling, stimulating tip cell formation in one cell while inhibiting neighboring cells from differentiating into tip cells. Notch also upregulates sVEGFR-1. The latter receptor traps VEGF and prevents excessive angiogenesis. The figure was generated by Biorender.

VEGFRs undergo alternative splicing to yield variants that regulate vessel homeostasis in various tissues [65]. VEGFR-1 mRNA undergoes alternative splicing to yield two variants, the membrane-bound VEGFR-1 and the soluble receptor VEGFR-1 (sVEGFR-1) [33]. Interestingly, VEGF indirectly regulates its own levels through the VEGFR-1 variant [34,35]. The binding of VEGF to VEGFR-2 activates Notch signaling, which ultimately leads to the differentiation of a select EC into a tip cell. In contrast, neighboring ECs form stalk cells of the new vessel sprout as they are prevented from forming tip cells [66,67,68,69]. VEGF/VEGFR-2 activates the protein kinase C (PKC)-MEK pathway, which triggers VEGFR-1 mRNA splicing and results in the formation of sVEGFR-1 (Figure 2) [67,68]. The formation of sVEGFR-1 is due to the splicing of mRNA at intron 13, which lacks the seventh immunoglobulin-like domain and the intracellular kinase domain [67,68]. Simultaneously, activated Notch induces sVEGFR-1 expression in ECs [68]. The soluble receptor binds VEGF with high affinity and, due to its lack of the kinase domain, cannot transduce intracellular signals efficiently [67]. Since sVEGFR-1 binds to VEGF, the levels of VEGF that are available to bind to VEGFR-2 become diminished (Figure 2) [13,66]. Notably, sVEGFR-1 can also bind PlGF and VEGF-B and sequester them [67].

Since sVEGFR-1 is important in vascular patterning, its deficiency impairs vessel structure [67,70]. The physiological function of sVEGFR-1 in vascular patterning is similar to that of VEGFR-1 in so far as it sequesters VEGF [71]. Since sVEGFR-1 sequesters ligands that largely play a role in pathological angiogenesis, PlGF, and VEGF-B, it may have application in PC treatment.

Alternative splicing of the terminal exons of the VEGFR-3 gene produces both membrane-anchored and soluble receptor isoforms [66,72]. The soluble transcript, sVEGFR-3, binds the ligands VEGF-C and -D [72]. However, sVEGFR-3 limits the availability of the ligands and prevents them from binding to the membrane-anchored receptor [73,74]. The mechanism by which the former isoform exerts its physiological effects still needs to be determined. It is noteworthy that its aberrant expression has been identified in cancer [65,66,67,68,69,73,74]. Moreover, the clinical application of this soluble isoform in various tumors has been under active investigation and may have a role in PC therapy, particularly given the limitations of VEGFR-targeted treatments in this disease.

### 4.2. Potential Role of VEGFR Splice Variants in Pancreatic Cancer Therapy

Some splice products of VEGFRs were recently found to have clinical applications in cancer treatment (Table 2) and are being explored as valuable biomarkers for cancer diagnosis, prognosis, or treatment response [65,66,67]. Treatment strategies employing VEGFR splice products include sVEGFR-1 and sVEGFR-2, which abrogate pathways in angiogenesis and tumor cell growth. As mentioned earlier, sVEGFR-1 targets angiogenesis and inflammation by sequestering VEGF-A and PlGF, reducing their availability for signaling through membrane-bound receptors. Through this mechanism, sVEGFR-1 attenuates VEGF-VEGFR2-mediated angiogenesis and modulates PlGF/VEGFR-1 signaling, which influences macrophage activation and migration. While these effects have been documented in various cancer types, they underscore sVEGFR-1’s potential relevance in PC therapy by altering both the vascular and immune microenvironments [66]. Supporting this, preclinical studies have demonstrated that adenoviral vectors encoding soluble VEGFR-1 can effectively inhibit tumor growth and/or metastasis in PC mouse models [22]. Other strategies employ sVEGFR-3, which has been shown to play a critical role in lymphangiogenesis (Table 2) [66]. The potential of these splice variants lies in their ability to normalize the tumor vasculature, enhance drug delivery, and reduce metastasis. Although these VEGF-targeting therapies have been primarily studied in other malignancies, their potential applicability to PC is plausible and warrants exploration.

#### 4.2.1. sVEGFR-1 Antibodies Have Potential Therapeutic Application

Antibodies against sVEGFR-1 were shown to inhibit angiogenesis in experimental models of cancer [66,83]. Histological analyses of the tumor biopsies revealed fewer VEGFR-1-positive cancer cells, M2 macrophages, and myeloid progenitor cells compared to untreated controls [66,83]. It could be postulated that the mechanisms of anti-angiogenic action are attributable to (i) the inability of PlGF to bind to the receptor, which would, in turn, hamper (ii) the suppression of the migration of myeloid progenitor cells into the tumor microenvironment and the infiltration of the TME by M2 macrophages, as well as (iii) the reduction in VEGFR-1 expressing cancer cell survival. sVEGFR-1 has a formidable inhibitory effect on angiogenesis and was shown to be therapeutically effective in reducing tumor growth in pre-clinical murine cancer models [75,76]. Clinical studies have explored the role of sVEGFR-1 in PC, demonstrating its ability to inhibit tumor growth. Elevated levels of sVEGFR-1 have been observed in patients with PC compared to healthy controls, with these higher levels being associated with slower disease progression, suggesting a protective role for this soluble receptor in PC. Conversely, reduced levels of sVEGFR-1 have contributed to the aggressive nature of other cancers, highlighting the importance of sVEGFR-1 in modulating tumor behavior across malignancies [83,84]. Given the toxicity of multi-targeting RTKIs, it may be more beneficial to consider anti-sVEGFR-1 antibodies as a strategy for suppressing angiogenesis. In addition to sVEGFR-1, the soluble isoforms of VEGFR-2 and VEGFR-3 have been explored for cancer therapy.

#### 4.2.2. Role of sVEGFR-2 in Pancreatic Cancer Treatment

Soluble VEGFR-2 (sVEGFR-2) functions as a decoy receptor, sequestering VEGF-A and preventing its interaction with membrane-bound VEGFR-2, thereby impairing angiogenesis and endothelial cell proliferation. In PC, elevated levels of sVEGFR-2 have been reported, demonstrating superior diagnostic accuracy compared to VEGF alone [84]. Therapeutically, VEGF-Trap, a soluble VEGFR, has shown promise in preclinical models by reducing microvessel density, tumor growth, and metastasis in PC [28]. These findings suggest that targeting sVEGFR-2 could be critical in refining diagnostic and therapeutic approaches for PC.

#### 4.2.3. Potential of sVEGFR-3 in Cancer Treatment

A combination of sVEGFR-2 and sVEGFR-3—AAV8 administered as gene therapy reduced the incidence of metastasis in a murine ovarian cancer model during pre-clinical testing [78,79]. In a mouse model of endometrial cancer, sVEGFR-3 reduced lymphangiogenesis and tumor growth [74]. Noteworthy is that a vector expressing the soluble form of the receptor inhibited lymphangiogenesis and reduced lymph node metastasis in a mouse breast cancer model [80]. Shibata et al. employed soluble VEGFR-3 as a decoy receptor for VEGF-C/-D binding, where it was found to inhibit lymphangiogenesis and reduce lymph node metastasis in breast cancer-bearing mice [81]. Downstream, the inhibition of these growth factors is associated with the suppression of Janus kinase (JNK), ERK 1/2, and PKB, ultimately resulting in the inhibition of lymphangiogenesis [80]. The effectiveness of sVEGFR-3 Ig Fusion protein has been investigated and found to effectively reduce tumor lymphangiogenesis and macro metastasis in mouse xenografts of human hepatocellular carcinoma [82]. The application of sVEGFR-3 in drug design has recently received considerable attention [80,81,82], and further research is sure to shed light on the potential use of this soluble protein in cancer treatment in patients.

### 4.3. Proposed Approaches to Improve VEGFR Targeting in Pancreatic Cancer Treatment

**Figure 3:**
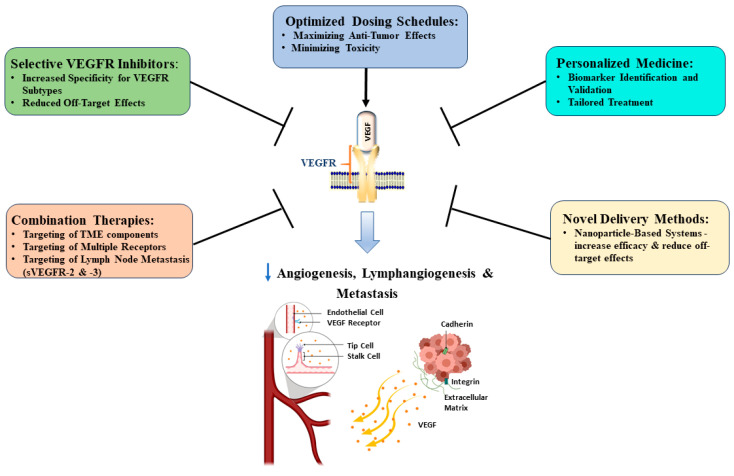
Proposed approaches to improve VEGFR targeting in pancreatic cancer treatment. The diagram illustrates the central role of VEGFR targeting and outlines several strategies to enhance its efficacy. Examples of ongoing clinical trials and specific drugs are highlighted to demonstrate the practical application of these strategies. The figure was constructed using drawing tools and Sketch.

Targeting VEGFR has emerged as a promising approach in cancer therapy, demonstrating significant potential in inhibiting tumor angiogenesis and growth [85]. However, despite its initial success in preclinical studies, the clinical efficacy has been disappointing [86,87,88]. This discrepancy highlights the challenges in translating laboratory findings to clinical success, possibly due to PC’s unique tumor microenvironment, characterized by dense, fibrotic stroma that may shield cancer cells from therapeutic agents [89]. Current efforts are focused on improving patient selection, identifying effective drug combinations, and enhancing the predictive value of preclinical models (Figure 3) [16,86,90].

One key area of focus is the development of more selective VEGFR inhibitors. Current inhibitors often exhibit off-target effects by inhibiting other kinases besides VEGF receptors, leading to side effects that may impact patient quality of life and reduce therapeutic efficacy. Designing molecules with increased specificity for VEGFR sub-types aims to minimize these off-target interactions, potentially resulting in improved safety profiles and enhanced antitumor activity. Combination therapies represent another promising avenue for improving VEGFR-targeted treatments. By pairing VEGFR inhibitors with other targeted agents or immunotherapies, the development of resistance could be curtailed, and therapeutic synergistic effects may be achieved. For instance, combining VEGFR and immune checkpoint inhibitors has demonstrated promising efficacy in preclinical studies, potentially enhancing the body’s natural immune response against cancer cells while simultaneously targeting the tumor vasculature [91]. For example, apatinib is currently being evaluated in a phase II clinical trial for treating metastatic or advanced PC in combination with the anti-PD-1 immune checkpoint inhibitor, camrelizumab [57]. Another promising therapeutic approach is to target multiple receptors simultaneously since PC expresses multiple angiogenic receptors [91]. However, one such therapy, regorafenib, did not show any benefit in a phase II clinical trial in refractory metastatic PC despite its preclinical efficacy and approval as a second-line treatment in metastatic colorectal cancer [26]. Also, pazopanib, a multitargeting tyrosine kinase inhibitor, showed modest antitumor activity as a combination therapy and indicated limited benefit as monotherapy in phase II clinical trials in patients with advanced PDAC and PNETs [92].

Novel delivery methods are also being investigated to optimize the efficacy of VEGFR-targeted therapies. Nanoparticle-based systems, in particular, have garnered significant attention. These advanced delivery platforms offer the potential to improve drug penetration into tumor tissues, enhance pharmacokinetics, and reduce systemic toxicity [93]. The encapsulation of VEGFR inhibitors within nanoparticles can achieve better-targeted drug delivery, potentially leading to improved therapeutic outcomes and reduced side effects [94]. Personalized medicine approaches are gaining traction in VEGFR-targeted therapies, departing from the “one size fits all” approach. Among the VEGFR subtypes, VEGFR-2 has been identified as a particularly critical target due to its central role in angiogenesis. However, VEGFR-1 and VEGFR-3 are also pivotal in precision medicine, with VEGFR-1 influencing tumor growth and immune modulation and VEGFR-3 playing a key role in lymphangiogenesis and metastatic dissemination [95]. Furthermore, ongoing research aims to elucidate the complex interactions between VEGFR signaling and the tumor microenvironment, highlighting the VEGF/VEGFR pathway as a significant regulator of the TME and supporting the potential benefits of combining VEGF/VEGFR inhibition with immune checkpoint therapy [86]. Additionally, the optimization of dosing schedules and treatment durations for VEGFR-targeted therapies can maximize their anti-tumor effects while minimizing toxicity [96].

## 5. VEGFR Role in Pancreatic Cancer Prognosis

Elevated VEGFR expression in PC is associated with enhanced tumor progression and poor overall survival, particularly involving VEGFR-2, which mediates angiogenesis and is linked to enhanced vascularisation and aggressive tumor behavior. Additionally, VEGFR-1 mobilizes bone marrow-derived cells and supports angiogenesis in hypoxic environments, whilst VEGFR-3 primarily facilitates lymphangiogenesis, promoting metastasis via the lymphatic system [97,98,99]. VEGFR activation stimulates endothelial cell proliferation, migration, and vascular permeability, thereby promoting tumor growth, invasion, and metastasis [100]. This signaling also contributes to chemoresistance, affecting treatment outcomes by triggering intracellular cascades, such as the PI3K/AKT and MAPK pathways, which promote cell survival and apoptosis resistance [101,102]. Consequently, PC cells become less susceptible to standard chemotherapies, leading to treatment failure and disease progression.

The complex interplay between VEGFRs and other signaling pathways exacerbates the aggressive nature of PC. VEGFR activation enhances matrix metalloproteinase (MMP) expression, promoting extracellular matrix degradation and tumor invasion [103,104]. Additionally, VEGFR signaling interacts with growth factor receptors like EGFR, amplifying pro-tumorigenic signals [80]. VEGFR-1 promotes cancer cell proliferation, migration, and invasiveness, while VEGFR-2/STAT-3 signaling facilitates stem cell renewal. Furthermore, VEGFR signaling induces immune tolerance by suppressing the TME, targeting various immune cells through VEGFR-1 (hematopoietic cells, macrophages, T-cells, and Treg cells), VEGFR-2 (T-cells and Treg cells), and VEGFR-3 (DCs and macrophages) [40,85,86,104].

VEGFR-3’s role in lymphangiogenesis increases the likelihood of lymph node metastasis through the HIF-1α/VEGF-C/VEGFR-3 axis [105]. Signaling through the VEGF receptors within the PC microenvironment signifies a complex regulatory network governing angiogenesis and tumor progression [16,31,106]. Thus, biomarkers that enable monitoring of the PC subtypes when targeting these receptors and employing molecules that regulate their splice products are crucial.

### Potential Biomarkers for Monitoring Treatment Effectiveness

Several potential biomarkers have been identified to monitor the effectiveness of VEGFR-targeted therapies in PC. The primary goal is to identify patients most likely to benefit from these treatments. However, it is essential to distinguish these biomarkers based on their roles in the prediction, pharmacological response, and disease prognosis. Although circulating VEGF levels have been investigated as potential biomarkers for assessing treatment effectiveness, limited evidence supports their prognostic significance, especially concerning VEGFR-targeted therapies. Most clinical trials have not consistently demonstrated that alterations in circulating VEGF levels reliably correlate with treatment outcomes [96]. In a phase II clinical trial where a combination of gemcitabine, cisplatin, and bevacizumab was used to treat PC, increases in circulating levels of VEGF were noted. However, the observed increase in circulating VEGF levels following treatment with bevacizumab was not significant [107]. Plasma sVEGFR levels, on the other hand, were shown to provide insight into the degree of VEGFR inhibition, with fluctuations in sVEGFR levels indicating the efficacy of anti-VEGFR therapies. Murukesh et al. describe soluble forms of VEGF receptors, sVEGFR-1 and sVEGFR-2, as decoy receptors that bind VEGF, preventing its interaction with cell surface receptors and inhibiting angiogenesis [96].

Changes in plasma levels of these soluble receptors can indicate the degree of VEGFR inhibition. For instance, decreased sVEGFR-2 levels are commonly associated with effective inhibition of VEGFR-2 signaling, crucial for endothelial proliferation and new blood vessel formation. Similarly, reductions in sVEGFR-3 may reflect lymphangiogenesis inhibition, although its correlation with clinical outcomes remains less well established [96]. Moreover, according to Chang et al., elevated serum levels of VEGF and sVEGFR1 correlate with worse survival in patients with PC, indicating their potential use as prognostic biomarkers for the disease [83]. Essentially, monitoring changes in sVEGFR levels provides insights into VEGF pathway engagement, with decreased sVEGFR-2 levels being the most consistent marker of therapeutic efficacy [96].

Circulating endothelial cells (CECs) are another potential biomarker whose increased levels may reflect ongoing angiogenesis or vascular damage, potentially indicating tumor response to VEGFR-targeted treatments [96]. It is posited that, generally, VEGF inhibition reduces CEC concentrations. However, some studies have displayed the opposite effect, with increased CECs being associated with observed improved outcomes. Despite this, methodological challenges exist in using CECs as competent biomarkers in PC. Similarly, circulating tumor cells (CTCs) can offer insights into treatment effectiveness through changes in their VEGFR expression patterns, with studies showing that VEGFR expression in CTCs is associated with metastatic potential and disease progression. Kallergi et al. noted that a significant proportion of CTCs in patients with metastatic breast cancer expressed VEGF, VEGFR-2, HIF-1α, and pFAK [108]. Notably, VEGF expression was detected in 73% of CTCs, whilst VEGFR-2 was present in 71%. These findings indicate that VEGF/VEGFR signaling likely plays a crucial role in CTC survival and metastatic potential. Tracking alterations in these expression patterns may serve as a potential indicator of treatment efficacy [108].

MicroRNAs (miRNAs) such as miR-126 and miR-200, involved in VEGF signaling, can serve as non-invasive biomarkers by reflecting changes in VEGF-related pathways during treatment. While most studies show miRNAs suppressing VEGF, one study found miR-126 positively correlated with VEGF expression in gastric carcinoma tissues [109]. This contradiction highlights the complexity of miRNA regulation in different cancer types. Therefore, imaging techniques that can facilitate the monitoring of treatment effectiveness in PC are necessary.

Imaging biomarkers, including dynamic contrast-enhanced MRI (DCE-MRI), have emerged as the most consistent biomarkers in trials of VEGF inhibitors, aligning with these drugs’ proposed mechanism of action. A reduction in transfer constants like K^trans^, which reflect vascular permeability and endothelial surface area, has been noted in patients treated with VEGF inhibitors. A 50% reduction in DCE-MRI parameters often correlates with stable or better disease outcomes, making it a promising prognostic and predictive biomarker for VEGF inhibitors that can be employed in the clinical management of PC. Furthermore, fractional plasma volume has been recommended as a useful marker in various clinical trials and may serve as an additional helpful biomarker in PC [96]. Tumor hypoxia markers, such as HIF-1α or CAIX, can indirectly assess the effectiveness of anti-angiogenic treatments by monitoring the increased hypoxia that results from reduced tumor blood supply, while Tie2-expressing monocytes (TEMs), involved in tumor angiogenesis, can be used to reflect anti-angiogenic therapy effectiveness by monitoring the changes in their levels or activation status [110,111].

Tissue-based biomarkers, such as microvessel density, pericyte coverage, and the expression of angiogenesis-related genes, provide direct evidence of VEGFR-targeted therapy’s impact on the tumor vasculature and also warrant exploration [96]. Once validated in clinical trials, these biomarkers could offer a comprehensive way in which to monitor the effectiveness of VEGFR-targeted therapies, guiding personalized treatment strategies for PC. Further research is required to determine the optimal combination of these biomarkers for improving clinical outcomes in PC management. Table 3 summarizes the advantages and limitations of these biomarkers, many of which have been studied in other cancers, with potential relevance to PC that warrants further investigation.

## 6. Conclusions

VEGFRs play critical roles in PC progression via angiogenesis and lymphangiogenesis. VEGFR-2, in particular, has been extensively studied because of its pivotal role in tumor vascularization and metastasis. However, current therapies targeting the VEGF/VEGFR-2 pathway, such as TKIs and monoclonal antibodies, have shown limited clinical efficacy in PC, primarily because of the emergence of resistance and the activation of compensatory pathways. Anti-VEGFR-2 drugs such as cediranib have shown limited effect even in combination with PD-L1 immunotherapy. VEGFR-1 and VEGFR-3, while less explored, are gaining attention for their roles in the modulation of the tumor microenvironment. Their distinct mechanisms underscore the need to address all three VEGFRs in tailored combination therapeutic approaches that target these VEGFRs and their signaling pathways to maximize therapeutic efficacy in PC. This review highlights the distinct roles of VEGFR-1, -2, and -3, their contributions to tumor progression, and the potential of novel inhibitors that selectively target these receptors. Additionally, the complex interplay between the VEGFRs and their splice variants highlighted herein emphasizes the need for a more comprehensive approach to designing targeted and selective therapies. Pre-clinical studies with sVEGFR-1 have shown promising results in PC as monotherapy and in other neoplasms as part of combination approaches. The identification and validation of predictive biomarkers remain essential for optimizing patient selection and enhancing the targeting efficiency of these treatments, as well as for monitoring therapy effectiveness in PC. By analyzing genetic profiles, protein expression patterns, and other proangiogenic molecular characteristics, clinicians may identify patients most likely to benefit from VEGFR-targeted therapies, thus tailoring an approach that could significantly improve treatment outcomes and resource allocation in cancer therapy. In addition, circulating VEGFR variants, circulating endothelial cells (CECs), and circulating tumor cells (CTCs) hold promise as biomarkers for assessing treatment response, reinforcing the need for biomarker-driven precision medicine strategies in PC.

## Figures and Tables

**Table 1:** VEGFR inhibitors that have been approved or are undergoing clinical development for PC treatment.

Drug	Target	Cancer	Status	Reference
Lenvatinib	VEGFR-1, -2, -3	Advanced-grade 1–2 PNETs	Phase II	[49]
Regorafenib	VEGFR-1, -2, -3; BRAF, cKIT, PDGFR-B	Refractory metastatic pancreatic cancer	Phase II	[50]
Cabozantinib	VEGFR-2	Advanced Pancreatic Neuroendocrine Tumors (PNETs)	Phase III	[51]
Cediranib	VEGFR-1 -,2, -3	PDAC	Phase II	[52]
Pazopanib	VEGFR-1, -2, -3, PDGFR, and c-Kit	PNET	Phase II	[53]
Sunitinib	VEGFR-1, -2, -3, PDGFR-α/β, c-KIT, FLT3 and RET	Locally Advanced or Metastatic PNETs	Approved 2011	[54]
Sorafenib	VEGFR-2, -3, PDGFR, c-Kit and RET	Advanced PDAC	Phase II	[55]
Axitinib	VEGFR-1, -2, -3	Advanced PDAC	Phase III	[56]
Apatinib	VEGFR-2	PDAC	Phase II	[57]

BRAF—v-Raf murine sarcoma viral oncogene homolog B, cKIT—Stem cell factor receptor (CD117), PDGFR-B—Platelet-derived growth factor receptor beta, PDGFR—Platelet-derived growth factor receptor, c-Kit—Stem cell factor receptor (CD117), PDGFR-α/β—Platelet-derived growth factor receptors alpha and beta, FLT3—FMS-like tyrosine kinase 3, RET—Rearranged during transfection.

**Table 2:** VEGFR variants and inhibitors exhibiting potential in cancer treatment.

Drug	Cancer	Effects	Reference
sVEGFR-1	Breast cancer PDAC	rAAV-sVEGFR1/R2 vectorVEGFR-1 binding and angiogenesis, suppresses macrophage infiltration, has anti-proliferative effect on cancer cells	[22,66]
sVEGFR-1-AAV 8	GBM	Inhibits angiogenesis and tumor growth	[75]
Recombinant sVEGFR-1 (rsVEGFR-1) + Carboplatin	Ovarian cancer	Inhibits ovarian cancer cell proliferation	[76]
rsVEGFR-1	Ovarian cancer, CRC	Has anti-proliferative effects on ovarian and colorectal cancer cells	[77]
sVEGFR-2 + sVEGFR-3—AAV8 gene therapy	Ovarian cancer	Reduces cancer metastasis, inhibits lymphangiogenesis	[78,79]
sVEGFR-3	Endometrial cancer	Reduces tumor growth, lymph node metastasis	[74]
sVEGFR-3 vector	Ovarian cancer	Decoy for VEGFR-3 binding to VEGF-C and -D	[80]
sVEGFR-3 gene therapy	Breast cancer	Inhibits lymphangiogenesis and multi-organ metastasis	[81]
sVEGFR-3-Ig Fusion protein	HCC	Inhibits tumor angiogenesis and lymphangiogenesis, suppresses primary tumor growth and lymph node metastasis	[82]

CRC—colorectal cancer, GBM—glioblastoma, HCC—hepatocellular carcinoma, PDAC—Pancreatic ductal adenocarcinoma.

**Table 3:** Advantages and limitations of potential biomarkers for VEGFR-targeted therapy in pancreatic cancer.

Biomarker	Advantages	Limitations	References
Circulating VEGF levels	Easily measurable in blood samples; consistent drug-induced increases in plasma VEGF-A levels across multiple studies	Lack of consistent prognostic or predictive value across studies; potential confounding factors in measurement, such as platelet activation during sample handling	[96]
Soluble VEGFR-1 and VEGFR-2	Reflects VEGFR inhibition; may indicate drug efficacy	Requires standardization; inconsistent correlation with outcomes	[83]
Circulating Endothelial Cells (CECs)	Can reflect vascular damage or active angiogenesis; some studies show correlations with clinical outcomes	Methodological problems in enumeration and characterization; lack of consensus on specific markers for CECs	[96]
Circulating Tumor Cells (CTCs)	Linked to metastatic potential and disease progression	Limited studies in pancreatic cancer; variability in VEGFR expression	[108]
MicroRNAs (miRNAs) (e.g., miR-126, miR-200)	Non-invasive; involved in VEGF regulation	Complex regulation; conflicting findings across cancer types	[109]
DCE-MRI (Imaging biomarker)	Non-invasive and sensitive detection method; shows consistent findings across multiple studies; Demonstrates dose-level response relationships; correlates with clinical outcomes in some studies	More complex to incorporate into multi-site studies compared to CT; requires standardization across different centers	[96]
Tumor Hypoxia Markers (HIF-1α, CAIX)	Enable indirect assessment of anti-angiogenic treatment effectiveness	May not be specific to VEGFR inhibition	[110]
Tie2-Expressing Monocytes (TEMs)	Reflect anti-angiogenic therapy effectiveness	Requires further validation in clinical settings	[111]
Tissue-Based Biomarkers (e.g., Microvessel density, Pericyte coverage)	Enable direct assessment of VEGFR-targeted therapy impact	Invasive, requiring tumor biopsies; lack of consistent predictive value for VEGF inhibitors; may not represent the entire tumor due to sampling limitations	[96]
